# Benzo[a]pyrene stress impacts adaptive strategies and ecological functions of earthworm intestinal viromes

**DOI:** 10.1038/s41396-023-01408-x

**Published:** 2023-04-17

**Authors:** Rong Xia, Mingming Sun, José Luis Balcázar, Pingfeng Yu, Feng Hu, Pedro J. J. Alvarez

**Affiliations:** 1 Soil Ecology Lab, Key Laboratory of Plant Immunity, Jiangsu Collaborative Innovation Center for Solid Organic Waste Resource Utilization and Jiangsu Key Laboratory for Solid Organic Waste Utilization, Nanjing, 210095 China; 2 Catalan Institute for Water Research (ICRA), 17003 Girona, Spain; 3 University of Girona, 17004 Girona, Spain; 4 College of Environmental and Resource Sciences, Zhejiang University, Hangzhou, 310085 China; 5 Civil and Environmental Engineering Department, Rice University, Houston, TX 77005 USA

**Keywords:** Metagenomics, Microbial ecology

## Abstract

The earthworm gut virome influences the structure and function of the gut microbiome, which in turn influences worm health and ecological functions. However, despite its ecological and soil quality implications, it remains elusive how earthworm intestinal phages respond to different environmental stress, such as soil pollution. Here we used metagenomics and metatranscriptomics to investigate interactions between the worm intestinal phages and their bacteria under different benzo[a]pyrene (BaP) concentrations. Low-level BaP (0.1 mg kg^−1^) stress stimulated microbial metabolism (1.74-fold to control), and enhanced the antiphage defense system (*n* = 75) against infection (8 phage-host pairs). Low-level BaP exposure resulted in the highest proportion of lysogenic phages (88%), and prophages expressed auxiliary metabolic genes (AMGs) associated with nutrient transformation (e.g., amino acid metabolism). In contrast, high-level BaP exposure (200 mg kg^−1^) disrupted microbial metabolism and suppressed the antiphage systems (*n* = 29), leading to the increase in phage-bacterium association (37 phage-host pairs) and conversion of lysogenic to lytic phages (lysogenic ratio declined to 43%). Despite fluctuating phage-bacterium interactions, phage-encoded AMGs related to microbial antioxidant and pollutant degradation were enriched, apparently to alleviate pollution stress. Overall, these findings expand our knowledge of complex phage-bacterium interactions in pollution-stressed worm guts, and deepen our understanding of the ecological and evolutionary roles of phages.

## Introduction

Earthworms are vital to soil ecosystems due to their widespread distribution and major contributions to the maintenance of the physical structure, drainage, nutrient availability, and microbial activity in soils [1]. Moreover, earthworms may improve bioremediation of contaminated soils by enhancing soil aeration through burrowing, and through enzymatic biotransformation of various contaminants, such as organic pollutants [2] and microplastics [3]. Recent advances in metagenomics analysis have shed light on the importance of the earthworm intestinal microbiome in the worm’s growth, behavior, and ecological roles [4–6]. The earthworm intestine is an anoxic microenvironment with abundant organic carbon and nitrogen, and is prone to accumulate soil contaminants [7, 8]. Therefore, to harness the full ecological and biotechnological benefits of earthworms, there is a critical need to advance our understanding of factors that shape their intestinal microbiome structure and functional diversity.

The virome contains the most abundant and genetically diverse biological entities in the biosphere [9]. There is an increasing recognition that the virome can substantially affect intestinal microbiome structure and functions through various phage-bacterium interactions [10–13]. Previous studies suggest that intestinal viromes are important reservoirs of bacterial metabolic genes and may modulate important microbial processes such as metabolic activities, quorum sensing, and chemotaxis pathways [14]. Other studies suggest that environmental stress such as drought [15] and pollutants [16] could affect the virome profile and modify the phage-bacterium symbiotic or antagonistic relationships. However, it remains largely unexplored how earthworm intestinal virome and phage-bacterium relationship responds to different environmental stress conditions. This represents a critical knowledge gap in exploring the roles of viromes in intestinal microbial homeostasis and in the adaptive mechanisms of earthworms facing various environmental stresses.

We used a multi-omics approach, including metagenomics, viromics, metatranscriptomics, and bioinformatics, to investigate the earthworm intestinal virome and its interaction with bacteriome under environmental pollution stress. Benzo[a]pyrene (BaP), a carcinogenic polycyclic aromatic hydrocarbon (PAH) was selected as a test soil pollutant because of its widespread presence and recalcitrance in soils [17] as well as its tendency to accumulate in earthworm gut [18, 19]. Multiple phage identification assays were adopted to characterize the earthworm intestinal phage community profile and its response to BaP-induced stress. Prognostic computational approaches were used for phage host prediction and to infer the phage-bacterium relationship. The phage-encoded AMG profile was also characterized to assess the potential of intestinal virome for microbiome resistance to environmental stress, which was further verified by metatranscriptomics. Overall, this study reveals adaptive strategies of intestinal viromes under BaP stress, and highlights the potential of phages to assist intestinal prokaryotic metabolism and resistance.

## Materials and methods

### Experimental design and BaP exposure

Surface farmland soil (depth 0–20 cm) without BaP contamination was collected from Nanjing, China (118°52’55” E, 32°2’21” N). The United States Environmental Protection Agency (USEPA) screening levels of BAP is between 0.1 and 1.8 mg kg^−1^ for resident soil [20]. Accordingly, in the exposure experiments, BaP (J&K Scientific; Guangdong, China) was amended to the collected clean soil at final concentrations of 0.1 (B1), 2.0 (B2), 20.0 (B3), and 200 (B4) mg BaP per kg soil, while soil without BaP contamination (CK) was used as the control. Based on BaP concentrations in soils, B1 was referred to as “low-level”, B2 and B3 as “medium-level”, and B4 as “high-level” BaP treatments, respectively. Five microcosms were prepared at each concentration following the standard process [21] and ten domesticated earthworms (*Metaphire guillelmi* from Yilong Earthworm Farm in Jiangsu, China) with clear clitellum and uniform body weight (~4 g each) were placed in each microcosm for BaP exposure. The microcosms with punctured lids were incubated at 20 °C in darkness for 28 days with a constant humidity of 15–20% [21]. The details of the microcosm preparation and exposure experiments are provided in the Supporting Information as Text S1 and Fig. S1.

After 28 days of incubation, earthworms were collected and the intestinal contents were obtained as previously described [22]. Briefly, the earthworms were anesthetized with anhydrous ethanol and rinsed with sterile water. The complete intestinal contents were collected by dissecting below the clitellum region using sterile scissors and scalpels. Intestinal contents were mixed and divided into several portions, which were stored at different temperature conditions for further analysis. Characterization of lethality and bioconcentration of BaP in the culture soil and earthworm gut after 28 days are shown in Table S1. The reactive oxygen species (ROS) content and the activity of antioxidant enzymes, including superoxide dismutase (SOD), catalase (CAT), and peroxidase (POD) in intestinal sample were determined to assess the response of earthworms to different levels of BaP. The details of the measurement are described in the Supporting Information as Text S2.

### Bacterial and phage DNA extraction, metagenome sequencing and assembly

Earthworm intestinal contents (0.25 g) from each treatment were collected for the extraction of total bacterial DNA using DNeasy PowerSoil Pro Kit (Qiagen, Germany). The qualified DNA was used for determining bacterial biomass in earthworm gut by quantitative PCR using universal primers targeting the V4 region of the 16S rRNA gene. Primer sequences were 515F (5’-GTG CCA GCM GCC GCG GTA A-3’) and 806R (5’-GGA CTA CHV GGG TWT CTA AT-3’). For phage DNA extraction, virus-like particles (VLP) in the earthworm intestinal contents were first purified and concentrated by filtration and centrifugation following the previously described protocol [23, 24]. Briefly, earthworm intestinal contents were suspended with 1% potassium citrate buffer (10 g l^−1^ C_6_H_5_K_3_O_7_, 1.92 g l^−1^ Na_2_HPO_4_·12H_2_O, 0.24 g l^−1^ KH_2_PO_4_, pH = 7), and then centrifuged at 7000 rpm 4 °C for 10 min to obtain the supernatant. The supernatant was filtered through 0.22 µm filter (Anpel hydrophilic PTFE syringe filter, China) and then concentrated by tangential flow filtration (TFF, Sartorius Vivaflow50 30000 MWCO PES, USA) [24]. Phages were precipitated using polyethylene glycol (PEG 8000, Biofroxx, Germany) and incubated in ice at 4 °C overnight, followed by centrifugation (8000 rpm for 30 min). Then VLP pellets were resuspended in TE buffer (Biosharp, China) and treated with DNase (TaKaRa Recombinant DNase I (RNase-free) 2270A) and RNase (Takara Ribonuclease A 2158). To determine the biomass of phages in the earthworm gut, fluorescence microscopy (Zeiss Axio Imager A1) was used to observe and quantify VLP stained with SYBR Gold fluorescent dye as previously described [16]. The ratio of VLP counts to bacterial 16S rRNA copy numbers was used to estimate virus-to-bacteria ratio (VBR). Thereafter, MagPure Viral DNA/RNA Mini LQ Kits (Angen Biotech Co., China) were used to extract phage DNA and the whole phage genome was amplified by REPLI-g Cell WGA & WTA Kit (Qiagen, Germany). The quality of phage DNA amplified products was verified by 1% agarose gel, Qubit 3.0, and Nanodrop One.

Both bacterial and phage sequencing libraries were generated using NEB Next Ultra DNA Library Prep Kit for Illumina (New England Biolabs, MA, USA) following the manufacturer’s recommendations. The libraries were then sequenced using the NovaSeq6000 system (Illumina, San Diego, CA) to yield 150 bp paired-end reads. This approach yielded 78,071,036–108,731,722 reads (average 99,860,922 reads/sample) for metagenome and 36,296,118–38,203,828 reads (average 36,739,983 reads/sample) for virome, respectively. Trimmomatic (v0.36) was used for raw data processing [25] and BWA-MEN (v0.7.17) was used to remove possible eukaryotic genome sequence with parameter -k = 30 [26]. Afterwards, the contigs from the bacterial and phage fractions were assembled separately using Megahit (v1.1.2) with parameters as: k-min 35, k-max 95, k-step 20 for bacteria and --presets meta-large, -min-contig-len = 300 for phage [27].

### Bacterial taxonomic assignment and functional annotation

MetaGeneMark (v3.38) [28] was used to predict open reading frames (ORFs) with length information shorter than 90 bp filtered out using default parameters. The CD-HIT (v4.7) [29] was adopted to remove redundancy and obtain the unigenes (i.e., the nucleotide sequences coded by unique and continuous genes) catalog with default parameter: -c 0.95 -aS 0.8. The longest sequences in each catalog were chosen to be the representative sequences. Then, the gene catalogs were mapped to clean data using BBMap (v38.90, https://github.com/BioInfoTools/BBMap) as default parameter in order to determine the abundance of genes in each sample. MetaPhlAn2 (v2.7.6, https://github.com/biobakery/MetaPhlAn2) [30] was used for the subsequent analysis of bacterial classification and abundance at both phylum and genus levels [31]. Functional genes were annotated by matching to functional gene database KEGG (Release 101.0) [32] and eggNOG (v4.5.1) using DIAMOND with e-value ≤0.001 [33].

### Phage contig identification for taxonomic assignment and functional annotation

CD-HIT (v4.7) was used to cluster the contigs longer than 5 kb of all samples to obtain unique phage contigs with parameter -c at 0.95 and -aS at 0.8. Two independent methods, VirSorter2 (v2.1.0) [34] based on gene enrichment and DeepVirFinder (v1.0) [35] based on designing convolutional neural networks to automatically learn phage genomic signature, were used to identify phage contigs comprehensively. The contig included in output file “final-viral-combined.fa” of virsorter2 and satisfied *p* ≤ 0.05 in DeepVirFinder were considered as phages. The phage contigs detected by one or both methods were classified by querying protein sequence against the HMM models with specific phage taxon from the ViPhOG database using “hmmscan” from HMMER (v3.1b2) [36]. The BLASTp mode incorporated in vConTACT2 (v0.9.17) [37] was used to complement phage classification and explore the overlap of earthworm gut phages with phage genomes from human gut [38], soil [39], honey bee gut [14], and Refseq [40] database utilizing genome gene-sharing profiles. The identified phage contigs were then further analyzed by BWA-MEN (v0.7.17) to compare clean reads and the reads per kilobase of exon model per million mapped reads (RPKM) value of each contig was calculated to obtain the phage abundance. Prodigal (v2.6.3) was used for gene prediction of phage contigs using the meta option [41] and DRAMV (v1.2.0) combined with VIBRANT (v1.2.0) was used for phage-encoded AMG identification with default parameters [42]. The possible function of AMGs was further supported by protein structure prediction. Phyre2 (v2.0) was used for secondary and tertiary structural homology searches for each AMG with alignment coverage above 70% and SWISS-MODEL server (https://swissmodel.expasy.org/, accessed on 11 December 2021) was then used to predict the quaternary structure of each protein with a Global Model Quality Estimation (GMQE) score above 0.5. Then BPROM server (http://www.softberry.com/, accessed on 2 January 2022) and FindTerm server (http://www.softberry.com/, accessed on 2 January 2022) were used to predict the promoter and terminator of AMGs, respectively [43].

### Phage-bacterium interaction analysis

Three prognostic computational approaches (CRISPR-match, tRNA match, and genome homology match) were adopted to improve the accuracy of phage host prediction [44]. Specifically, the bacterial scaffolds were used for searching CRISPR spacers by CRISPR recognition tool (CRT, v2.1) [45]. The identified CRISPR spacers were compared with phage contigs using BLASTn (v2.9.0+), and the thresholds of satisfying ≥95% identity and ≤2 single nucleotide polymorphisms (SNPs) were selected to serve as putative phage hosts. In parallel, tRNA genes in phage contigs were predicted by tRNAscan-SE (v1.23) with default settings and blasted against the bacterial sequences using the BLASTn, keeping only best hits with at least 95% sequence identity [46]. Moreover, phage genomic signatures in microbial genomes, lysogens, were identified via a search against the bacterial scaffolds through BLASTn with following parameters: bitscore ≥50, e-value ≥10^−3^, identity ≥70%, and matching length ≥2500 bp [47]. Phage hosts obtained by each method were combined to analyze the host range comprehensively given the high recognition of the three host prediction methods [44]. Phages linked to multiple bacterial genera were regarded as polyvalent phages (i.e., the phage able to infect diverse hosts) [48]. To better understand the variation in phage-bacterium association, DefenseFinder server (https://defense-finder.mdmparis-lab.com/, accessed on 13 August 2022) was used to identify antiphage defense systems in bacteria [49], which classified the defense mechanisms as previously suggested [50].

To assess the symbiosis of bacteria and phages in different treatments, the proportion of lysogenic phages in the gut virome and lysogenized bacteria in the gut bacteriome were separately identified. Specifically, lysogenic phages were identified by Deephage (v1.0) using 0.6 as the cutoff. The lysogenic marker proteins in phage sequence (transposase, integrase, excisionase, resolvase, and recombinase proteins downloaded from Pfam (v35.0); Table S10) were identified using “hmmscan” from HMMER (v3.1b2) with e-value threshold of 10^−5^ when the Deephage probability score was not deemed confident [51]. Additionally, prophages were identified in bacterial genomes according to known phage signatures with CheckV (v0.8.1) and Prophage hunter server (https://pro-hunter.genomics.cn/index.php/Home, accessed on 4 May 2022).

### Transcriptomic analysis of bacterial and phage functional genes

The transcriptome of earthworm gut microbiome was obtained to investigate the expression of bacterial genes and phage-encoded AMGs at different BaP levels. In brief, the earthworm gut contents were frozen in liquid nitrogen immediately after dissection. Then total RNA was extracted using RNeasy Mini Kit (Qiagen, Germany) from 30 mg sample, which quality and quantity were determined by Agilent 4200 system (Agilent Technologies, Waldbron, Germany) and NanoDrop2000 (Thermo Fisher, MA, USA), respectively. Whole mRNAseq libraries were generated using NEB Next Ultra Nondirectional RNA Library Prep Kit for Illumina (New England Biolabs) following manufacturer’s recommendations. The rRNA transcripts were eliminated by ALFA-SEQ rRNA depletion Kits (for Bacteria). The mRNA was converted into cDNA, followed by purification and amplification to obtain the sequencing library. Paired-end sequencing was conducted on a NovaSeq6000 system to generate 150 bp paired-end reads with low quality and short filtered with Trimmomatic (v0.36). Clean reads were mapped to NCBI Rfam (v14.9) databases to remove non-protein-coding rRNA sequences from eukaryotes and prokaryotes by Bowtie2 (v2.33). Afterwards, the remaining clean reads were assembled into a de novo transcriptome using Trinity (v2.4.0) with the parameters: --pre_correction --mink 20 --maxk 60 --step 10. Prodigal (v2.6.3) was used to predict ORFs for assembled Scaftigs longer than 200 bp and the unigenes were clustered using methods consistent with metagenomics [29]. BLASTx (v2.9.0+) was used to compare the unigenes with eggNOG (v4.5.1), CAZy (v2016.7.15), and KEGG (Release 101.0) database for functional annotation. Bowtie2 (v2.33) was used to align the clean reads back to the unigenes with default parameters for quantification of gene expression levels. The expression profiles of phage-encoded AMGs were also determined by mapping the clean metatranscriptomic reads to AMG sequences using BBMap (v38.70) with strict criterion (100% identity and 100% coverage) to avoid false alignment to bacterial transcripts [15, 52, 53]. The relative abundance of genes was determined by RPKM for normalization [53].

### Statistical analysis

The alpha diversity indexes of the microbes in each sample were determined using the “diversity” function in vegan package in R (v4.0.3). ANOSIM analysis was used to determine the significance of the differences in intestinal microbial communities. Differential expression (DE) of two treatments was estimated using “DEseq” function in DEseq package in R (v4.0.3). KEGG enrichment analysis of significantly augmented genes were implemented using Fisher’s Exact Test (https://www.omicshare.com/tools/). The significance of differences in parameters were compared using *t*-test in SPSS 26.0. Data visualization was performed in the website: https://www.chiplot.online/ and R (v4.0.3).

## Results and discussion

### BaP induced oxidative stress and altered the intestinal bacterial community

**Fig. 1:**
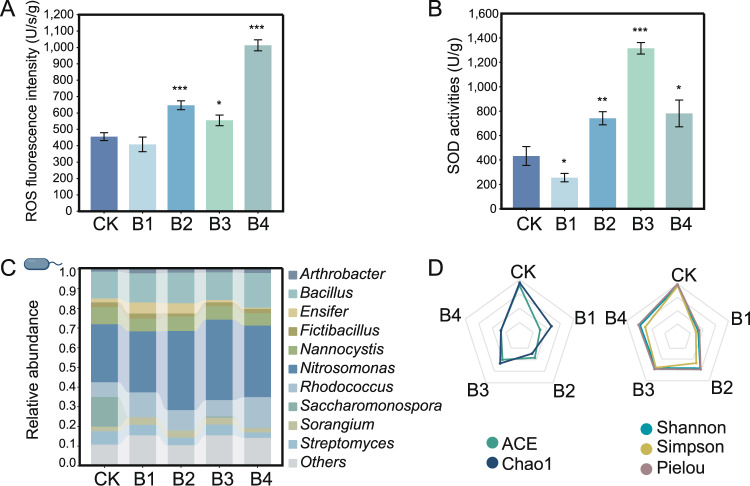
Toxicity of Benzo[a]pyrene (BaP) to earthworm intestines and gut bacterial community. Reactive oxygen species (ROS) (**A**) and superoxide dismutase (SOD) activity (**B**) of earthworm gut exposed to BaP at 0 (CK), 0.1 (B1), 2.0 (B2), 20 (B3), and 200 (B4) mg per kg soil. **C** Relative abundance of the top 10 abundant intestinal bacteria genus. **D** Alpha diversity (ACE and Chao1 indexes for richness, and Shannon, Simpson, Pielou indexes for evenness) of the bacterial community in the earthworm intestines. The radar map is drawn according to the data in Table S2 after normalization. The error bars in this study mean standard deviation of three replicates. *p < 0.05 indicates significant differences between the BaP exposure treatment and control, based on the *t*-test.

Although low-level BaP did not noticeably change ROS production, high-level BaP significantly induced ROS production in the earthworm gut. When earthworms were exposed to 2.0, 20.0, and 200 mg kg^−1^ of BaP, intestinal ROS concentrations increased by 1.42-, 1.22-, and 2.22-fold, respectively (Fig. 1A). In response to this oxidative stress, the intestinal intracellular antioxidant systems were stimulated (*p* < 0.05) as BaP concentrations increased (Figs. 1B and S2). SOD activity was 1.7- and 3.0-fold higher than that of control group when earthworms were exposed to 2.0 and 20.0 mg kg^−1^, respectively (Fig. 1B). However, excessive oxidative stress could deactivate antioxidant enzymes [54, 55] and result in a significant decrease in SOD activity, as observed under high-level (200 mg kg^−1^) BaP exposure (*p* < 0.05; Fig. 1B). Accordingly, the functionally active intestinal microbial profile altered significantly under BaP-induced stress (*p* < 0.05, Fig. S3). Particularly, bacterial genera with high environmental adaptivity (*Bacillus* and *Pseudomonas*) [56, 57] were enriched as the BaP levels increased (Fig. 1C). BaP exposure decreased the intestinal microbial diversity including community evenness (i.e., Shannon, Simpson, and Pielou indices) and community richness (i.e., Chao1 and ACE indices) (Fig. 1D and Table S2). In addition, we observed the lowest community evenness at low-level BaP concentration. However, these indices increased with the pollutant levels, which was consistent with the previous microbiome study [58].

**Fig. 2:**
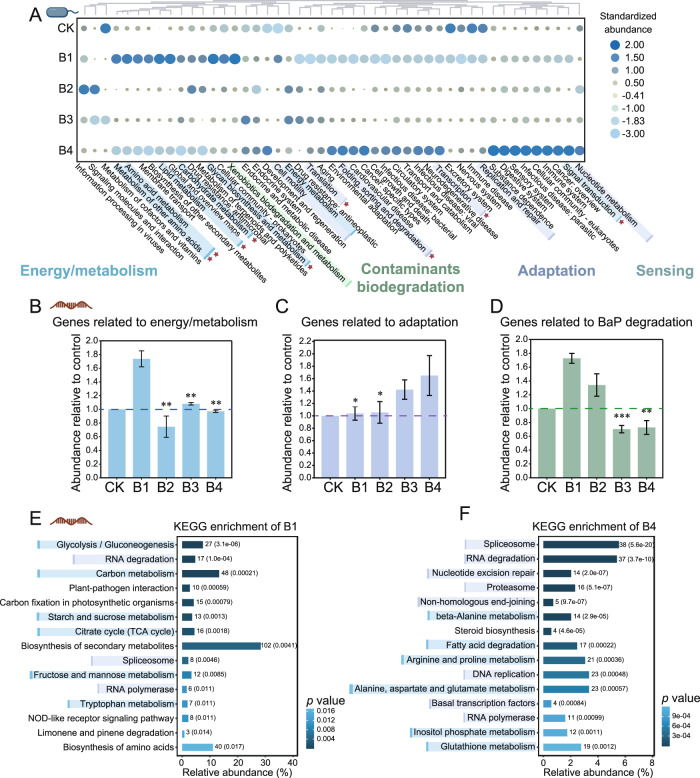
Functional profile and metatranscriptome validation of prokaryotic community in earthworm intestines. **A** Heatmap shows the relative abundance of KEGG pathway (Level 2) of earthworm intestinal prokaryotic community. Abundance is normalized among different treatments based on *Z*-score. Red stars represent pathways with significant differences in expression levels in metatranscriptome. The regulation of genes related to energy and metabolism (**B**), stress adaptation (**C**) and BaP degradation (**D**) in metatranscriptomes relative to the control group. The histogram shows the top 15 enriched KEGG orthology (KO) pathways of enriched genes under low- (**E**) and high- (**F**) level BaP exposure. The number of enriched genes in each pathway is labeled in front of the histogram, and the bars are ranked by *p* values. “Relative abundance” represents the proportion of significantly enriched genes to all genes in the KEGG pathway. The dark red single stranded nucleic acid shown in the diagram represents data obtained from the metatranscriptome.

Transcriptomic analysis corroborated the toxic effects of BaP on microbial functions inferred by metagenome sequencing. Low-level BaP exposure enriched the expression of genes coding for bacterial energy production pathways (e.g., carbohydrate, lipid, and amino acid metabolism) [59], but the relative abundance of these pathways decreased dramatically under high-level BaP exposure (Fig. 2A). Metabolic pathways closely related to bacterial survival (nucleotide metabolism and genome replication and repair) [60–62] and those associated with bacterial sensing (signal transduction) [63] were significantly enriched in the high-level group (Fig. 2A). Transcriptome analysis also corroborated that the genes associated with energy production were significantly enriched by low-level BaP exposure (1.74-fold) compared to the control (*p* < 0.05), but were significantly depleted when BaP increased further to 200 mg kg^−1^ (*p* < 0.05; Fig. 2B). However, the relative abundance of genes associated with microbial adaptation (e.g., *sbcB* involved in DNA repair and recombination and *XPF* encoding proteins for DNA excision repair) increased with BaP concentration and was 1.65-fold higher than that of control at 200 mg kg^−1^ BaP (*p* < 0.05; Fig. 2C). The functional genes that contributed to BaP degradation such as dioxygenase *hcaD*, dehydrogenase *XDH*, and hydrolase *mhpC* also enriched in low-level BaP exposure (1.72-fold higher than that of control, Fig. 2D).

KEGG enrichment analysis was conducted for the genes that were significantly enriched in each BaP concentration to explore the major metabolic pathways where these genes are located (Figs. 2E, F and S4A, B). Low-level BaP exposure significantly enriched genes in glycolysis/gluconeogenesis, carbon metabolism, starch and sucrose metabolism, and citrate cycle (TCA cycle), which are the pathways closely related to microbial energy production [64] (Fig. 2E). In contrast, enriched genes in the high-level BaP group were distributed in spliceosome, DNA damage repair, RNA degradation, proteasome, and other metabolic pathways related to microorganisms adaptation and survival [65] (Fig. 2F). Overall, metagenomic and metatranscriptomic analyses show that BaP-induced stress significantly affected the composition, diversity, and functional profile of the earthworm intestinal bacterial community, which is consistent with previous studies showing that earthworm gut microbes are sensitive to pollutant exposure [66]. As a microbial adaptive strategy to environmental stress, the enrichment in the functional genes that contributed to pollutant degradation and oxidative stress alleviation corroborated that microbes in earthworm gut could facilitate host earthworms to survive hostile environments (Fig. 2D).

### BaP exposure affected the composition and lifestyle of phage community in the earthworm gut

**Fig. 3:**
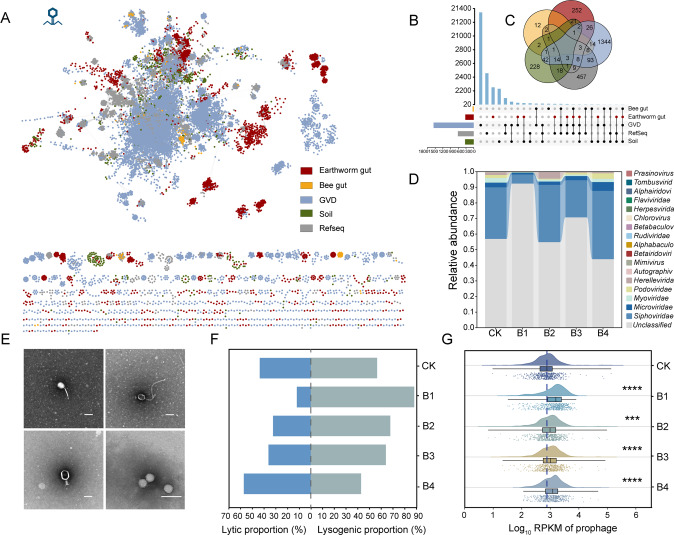
Composition and lifestyle of earthworm gut virome. **A** Gene-sharing network associates viral contig in earthworm gut (red nodes) with datasets of viral genomes that includes viral sequence from bee gut (yellow nodes), human gut (blue nodes), soil (green nodes), and RefSeq (gray nodes). The edges indicate similarity based on shared protein clusters. **B** The matrix layout shows the number of VCs that are exclusive (one circle) or shared (multiple circles) between the five different datasets used for clustering. **C** Venn diagram of shared VC among the five different datasets. **D** Community compositions (family level) of phages in earthworm gut. **E** Transmission electron microscopy (TEM) image of typical phage in earthworm intestines. The scale bars shown in the figures are 100 nm. **F** Proportions of lytic phage and lysogenic phage in earthworm gut. **G** Abundance (Log10 RPKM) of prophage.

A total of 2629 non-redundant phage contigs larger than 5 kb were identified from all the five groups of earthworms intestinal viromes (Table S4). These intestinal phages formed 325 viral clusters (VC) using the existing phage datasets and presented high similarity to human gut and soil phages (Fig. 3A–C). Among them, a total of 41% phage contigs could obtain species annotation information. Taxonomic classification suggested that the dominant taxa in earthworm gut virome include *Siphoviridae*, *Microviridae*, *Myoviridae*, and *Podoviridae* (Fig. 3D), which was corroborated by morphology analysis using TEM imaging (Fig. 3E). When earthworms were exposed to 0, 0.1, 2.0, 20.0, and 200 mg kg^−1^ BaP, the relative abundance of *Siphoviridae* were 33.1%, 5.8%, 36.6%, 23.8%, and 43.7%, *Microviridae* were 3.0%, 1.2%, 2.2%, 2.7%, and 5.7%, and *Myoviridae* were 3.0%, 0.2%, 1.3%, 0.9%, and 2.0%, respectively (Fig. 3D). The species diversity of phage community was the lowest under low level BaP exposure, and then increased along with the elevated BaP concentrations (Table S5). This could be caused by the activation of prophages and the release of virions under high-level BaP exposure.

BaP exposure remarkably affected the lifecycles of intestinal virome. Lysogenic phages accounted for 56%, 88%, and 43% of total phages in control, low-level BaP, and high-level BaP exposure, respectively (Fig. 3F). Similarly, the relative abundance (average log10 RPKM) of prophages also exhibited bell-shaped stimulatory effect, which was the highest in low level BaP (3.10, *p* < 0.05) (Fig. 3G), and was consistent with the relative abundance of lysogenic bacteria (Fig. S5). Moreover, evidence from the metatranscriptome sequencing also showed that lysogenic marker genes (transposase, integrase, excisionase, resolvase, and recombinase) were significantly enriched under low-level BaP exposure, indicating that phages were prone to integrate into bacterial genome and became lysogenic phages in worm gut at low BaP level. But the relative abundance of these lysogenic marker genes decreased substantially under high-level BaP exposure (Fig. S6), suggesting the decrease in lysogenic phages. These results suggest that phages adopted a lysogenic adaptive strategy at low BaP levels and converted toward a lytic lifestyle at high BaP levels. High-level BaP, similar to other environmental stressors (e.g., high temperatures, nutrient depletion, and anomalous pH) [67, 68], can induce prophages conversion to a lytic lifecycle. The significant enrichment of SOS response genes *dinF* and *ssb* [69] and depletion of repressor gene *lexA* [70] indicate that high-level BaP exposure could activate the SOS system and trigger a phage lifestyle switch from lysogenic to lytic (Fig. S7).

### Phage-encoded AMGs were associated with PAH degradation and antioxidation

**Fig. 4:**
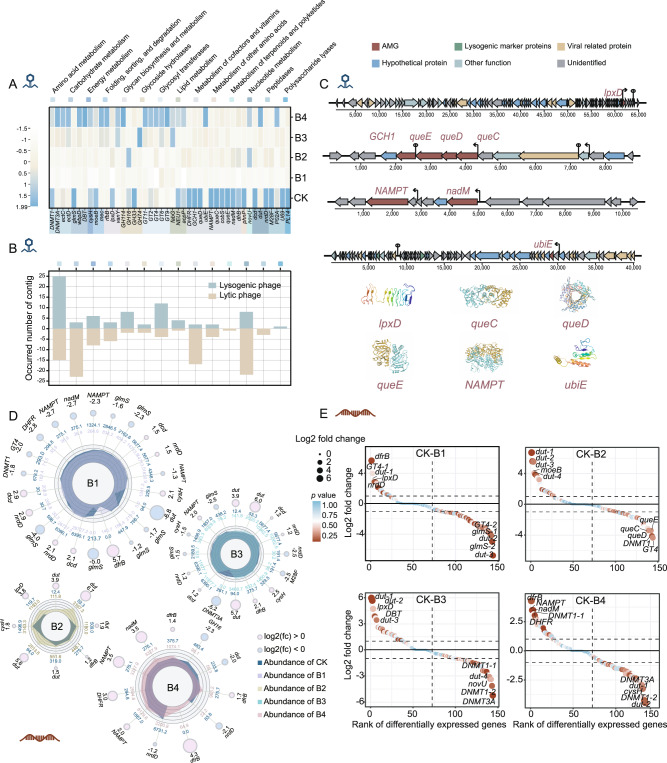
Phage-encoded auxiliary metabolic genes (AMGs) and their transcriptome profiles under BaP stress. **A** Heatmap shows the classification of AMG in KEGG level 2 pathways and their relative abundance. **B** Number of AMGs in lysogenic and lytic viral contig respectively. “Occurred number of contig” means the number of contigs carrying a certain class of AMGs in different lifestyles. **C** Genomic context and protein structure of six phage encoded AMGs associated with BaP resistance and degradation. **D** Significantly up-regulated or down-regulated gene abundance in different samples and its log2 fold change (fc) compared with the control. When log2 (fc) >0 represents that the gene in the sample is significantly up-regulated compared with control, and vice versa. **E** Rank the differentially expressed genes according to the log2 fold change. Each circle represents a gene, and the color of the circle represents the significance of the difference in gene expression. The genes annotated to the same function were distinguished by different symbols with detailed information listed in Table S8.

To understand the role of intestinal phages in alleviating BaP stress, we investigated the diversity and functions of phage-encoded AMGs in earthworm gut. A total of 203 phage AMGs encoding 44 types of metabolic functions were identified through VIBRANT and DRAMV analysis (Fig. 4A and Table S6). The most common AMGs in lysogenic phages were associated with amino acid metabolism, while AMGs associated with nucleotide metabolism were relatively more abundant in lytic phages (Fig. 4B). These AMGs involved in carbohydrate, glycan, amino acid, and sulfur transformation, which have been widely recognized in marine, terrestrial, and other environments [39, 43, 71], were conductive to microbial metabolism and energy production. Several AMGs, such as *dcd*, *dut*, and *nrdD* that are related to pyrimidine metabolism are known to promote bacterial genome replication and reparation [72] (Fig. 4A). Three AMGs associated with cell membrane integrity and robustness were detected for the first time in the viromes, including *lpxD*, *vanY*, and *NEU1* that were related to lipopolysaccharide biosynthesis, peptidoglycan biosynthesis, and sphingolipid metabolism, respectively [73, 74] (Fig. 4A, C). Additionally, 19 novel AMGs were associated with biosynthesis of folate (such as *dfrB*, *queC*, *queD*, and *queE etc*.), which has potent antioxidant properties that alleviate ROS stress [75]. Among them, *queC*, *queD*, and *queE* were located in the same genomic neighborhood and shared the same promoter and terminator (Fig. 4C), indicating that the functions of these three phage-encoded AMGs were complementary [76]. These AMGs that are associated with microbial adaptation were enriched as BaP stress elevated (Fig. 4A). Notably, AMGs for PAH biodegradation (*ubiE*) [77] were also identified and their relative abundance increased by 55.4-fold in the high-level BaP group (Fig. 4A, C). The structural model prediction of phage-encoded *ubiE* at Phyre2 showed 99.8% confidence, suggesting the AMG sequence could encode a structurally intact protein and perform its function (Fig. 4C and Table S7). This is the first detection of *ubiE* as a phage-encoded AMG, which encoded benzoquinol methylase to catalyze the anaerobic degradation of phenanthrene [61].

Transcriptomic analysis further confirmed that phage-carried AMGs could facilitate microbial resistance to BaP-induced stress. Among them, 103, 113, 116, and 115 AMGs were expressed when BaP exposure levels were 0.1, 2.0, 20.0, and 200 mg kg^−1^, respectively (Fig. S8A). The corresponding sum expression abundance (RPKM value) of these AMGs were 1.16E + 05, 5.87E + 04, 8.55E + 04, and 9.19E + 04, respectively (Fig. S8B). Compared to the control group, *ubiE* was enriched by 1.35- and 5.17-folds at 0.1 and 2.0 mg kg^−1^ BaP exposure, respectively (Fig. S8A). Furthermore, *lpx*D related to microbial resistance to BaP was enriched by 14.1 folds when earthworms were exposed to 2.0 mg kg^−1^ of BaP (*p* < 0.05, Fig. 4D). In addition, genes associated with microbial resistance to oxidative stress (e.g., *dfr*B, NAMPT, and *nad*M [78, 79]) were significantly enriched under BaP-induced stress (*p* < 0.05, Fig. 4D, E).

With the expression of approximately half of the annotated AMGs in viromes, the transcriptomic results verified the important roles of these AMGs in mediating bacterial metabolism in worm gut, especially bacterial resistance to BaP stress. Those AMGs that were not detected in the transcriptome could still serve as functional repositories and play an ecological role in the microbial community through horizontal gene transfer (HGT) [14]. To capture the temporal characteristic of AMG expression, it is important to apply multi-omics analysis to comprehensively reveal the influence of different stress on phages activity, contribution of phages to host bacterial metabolism, and the interactions between phages and bacteria [15, 52, 80]. Although omics analysis greatly advances our understanding of unculturable microbes and their interactions, microbial experiments with representative culturable species can often provide more insights into the microbial processes and underlying mechanisms [80].

### BaP contamination affected phage-bacterium interactions and phage adaptive strategies

**Fig. 5:**
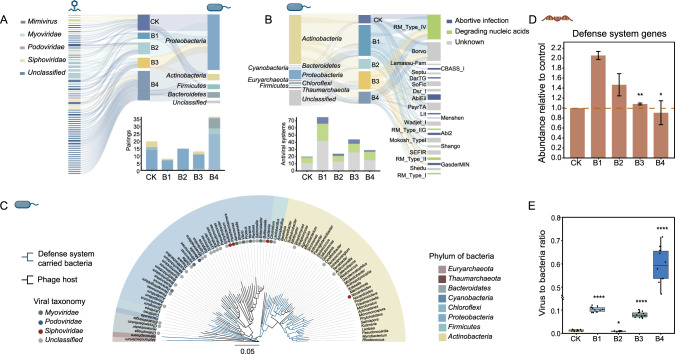
Analysis of phage-bacterium interactions. **A** Predicted phage-host associations. Left: the viral contigs that can match to host. Different colors present different taxonomic classification at the family level. Middle: the number of phage-host pairings in different samples. Right: the distribution of bacterial hosts at the phylum level. The bar chart below counts the number of pairings in each sample, and the classification of the different hosts in the bar is consistent with the information of the hosts on the right. **B** Identification of antiviral defense systems in bacteria. Left: taxonomic classification of defense systems carrying bacteria at the phylum level. Middle: the number of defense systems in different samples. Right: the subtype and the defense mechanisms of the defense system. The bar charts below show the number of antiviral defence systems. **C** The phylogenetic tree of the phage host and the bacteria that carry the antiviral defense system. **D** The expression abundance of defense system genes in metatranscriptomes are presented by the abundance relative to control. **E** Phage-to-bacterium ratio in earthworm intestines at different concentrations of BaP.

To elucidate the response of phage-bacterium interactions to BaP stress, we investigated putative phage-host linkages and antiphage defense systems carried by bacteria and VBR across all the intestinal samples. Based on the CRISPR-match, tRNA match, and genome homology match approaches, we could link 66 phage contigs distributed in *Mimivirus*, *Myoviridae*, *Podoviridae*, and *Siphoviridae* to 4 host phyla and 37 genera (Table S9). Putative host bacteria were *Proteobacteria*, *Actinobacteria*, *Bacteroidetes*, and *Firmicutes*, which were the dominant members in the bacterial community (Figs. 5A and S2C). A total of 93 phage-host pairs were matched in the five treatments of these 66 contigs, with more pairings were predicted as BaP stress elevated (*n* = 8 and 37 at low and high BaP exposure, respectively) (Fig. 5A). These results suggest that high-level BaP exposure resulted in more phage-host associations relative to low-level BaP exposure. More phages-host pairings indicated the increased infectivity of phage. Given the increased number of free phages, we can infer that the bacteria were subjected to greater phage predation pressure at high BaP concentrations. The abundance and diversity of antiphage defense systems carried by bacteria (and thus phage-bacterium interactions) changed as BaP stress increased. Specifically, 192 defense systems were observed in 55 bacterial genera in all treatments, corresponding to 22 subtypes and 2 known defense mechanisms “phage nucleic acid degradation” and “abortive infection” (Fig. 5B). The restriction-modification system (RM system), which protects against phage infection via nucleic acid degradation [81], was the most abundant defense system in all samples. Moreover, the most abundant and diverse defense systems were observed at low-level BaP exposure, possibly due to relatively high metabolic ability of bacteria. The largest number of defense systems were observed under low level BaP exposure (*n* = 75), which was consistent with the least phage-bacterium association in all samples. As expected, the bacteria carrying the dominant antiphage defense systems were mainly distributed in the phylum *Actinobacteria*, which was different from the predicted dominant prokaryotic hosts (Fig. 5C). The relative abundance of expressed antiphage defense system genes was also found with the highest abundance at low-level BaP exposure while lowest abundance occurred with high-level BaP exposure (Fig. 5D). This was consistent with the increased proportion of prophages at low-level BaP exposure, as non-essential transcription regions within the prophages genome can encode defense systems to prevent host bacteria from subsequent infection [82, 83]. In addition, the strong metabolic capacity of bacteria under low-level BaP exposure also enhanced the expression of prophages and antiphage defense systems simultaneously (Figs. 2B, D and S6) [84]. When the concentration of BaP increased from low to high level, the bacterial defense capability against phage infection and lysis became weaker. The active antiphage systems under low-level BaP exposure could prevent phages from successfully infecting bacteria, which explained the lower phage-bacteria linkages comparing with high-level BaP exposure.

**Fig. 6:**
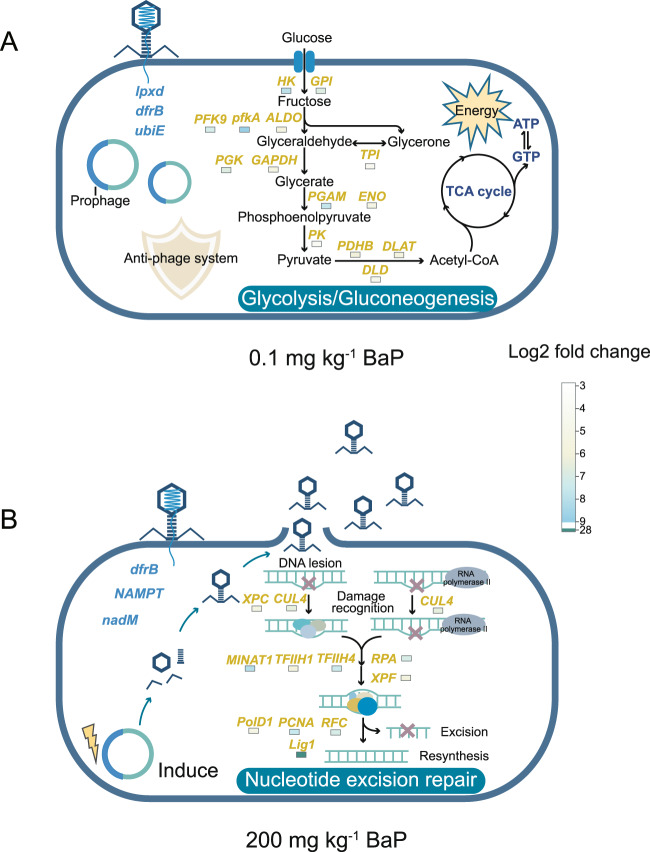
Conceptual depiction of metabolic functions of bacteria and phages under different BaP stress. The pathways “glycolysis/gluconeogenesis” and “nucleotide excision repair” were respectively taken as examples to emphasize the differences in physiological metabolic states of bacteria under low (**A**) and high (**B**) BaP concentration conditions. The yellow fonts represent genes that are significantly enriched in the transcriptome, and the color squares below the genes represent the log2 fold change compared to the control group. The blue fonts represent phage-encoded AMGs that are prominently expressed.

VBR was also determined to explore phage-bacterium interactions. The VBR was 0.01, 0.1, and 0.6 in clean, low, and high BaP contamination, respectively (Figs. 5E and S9 and S10), indicating that high level of BaP could result in the release of virions from bacteria and the phages adopted an adaptive strategy hostile to the host bacteria. At low BaP concentration, the bacterial density was relatively high and the phage was inclined to lysogenic lifestyle, while the phage lifestyle changed to lytic at high BaP concentration and lower bacterial quantity (Figs. 3F and S10). This switching indicated that the phages in the earthworm gut exposure to BaP were more inclined to adopt the Piggyback-the-Winner (PtW) life strategy, where phages tended to integrate into the host genome instead of lysing the hosts for replication despite of high host abundance. This resulted in a reduced release of virions and ultimately a low VBR [85, 86]. Under pollution stress, this life strategy has profound significance for the adaptation of bacteria and phages. When adopted a lysogenic lifestyle, the phages replicate by taking advantage of the high metabolic capacity of the bacteria at low-level BaP, which is favorable for progeny reproduction [87]. Prophages would protect their host from reinfection through superinfection exclusion mechanism, which may explain the decreased phage-host associations observed at low-level BaP [86, 88]. To alleviate metabolic burden associated with foreign genome integration, the prophages carried functional genes that could enhance host bacterial fitness (Fig. 6A) [14, 56, 58, 59]. However, when exposed to high-level BaP, the intracellular environment of the bacteria was no longer suitable for sheltering lysogenic phages; and prophages were induced and released into the environment via lysing the host bacterial cells, leading to the phage-bacterium interaction converting from mutualism to antagonism (Fig. 6B) [89]. Similar conversion was observed in the human gut when inflammation occurred and intestinal oxidative stress increased [90].

## Conclusions

We propose that phage-bacteria interactions and phage-encoded AMGs play important roles in facilitating microbial adaption to BaP stress in earthworm intestines. Low-level BaP stress can stimulate microbial metabolism through exerting a bell-shaped stimulatory effect in earthworm intestinal bacterial community, which enhanced the antiphage defense system and cooperative interaction between phages and bacteria. In contrast, high-level BaP exposure damaged bacterial metabolism and the antiphage systems, resulting in the shift from cooperative to antagonistic relationship between phages and bacteria. Despite the fluctuating interactions between phages and bacteria, the active expression of phage-encoded AMGs related to pollutant resistance and degradation could assist the host bacteria in alleviating pollution stress. Collectively, these results reveal the PtW life history strategy adopted by the earthworm intestinal phages under BaP exposure, and demonstrate the important ecological functions of intestinal phages in assisting symbiotic bacteria to survive pollution stress.

## Supplementary information

Supporting texts 1-2, Supplementary figures 1-10

Supplementary table 1-10

## Data Availability

All raw sequence data generated in this research have been deposited in the NCBI’s Sequence Read Archive (SRA) database and can be accessed under project accession no. PRJNA922612. All data are publicly accessible and can be download from https://www.ncbi.nlm.nih.gov/sra/PRJNA922612.
