# Supplementary material for: Benzo[a]pyrene stress impacts adaptive strategies and ecological functions of earthworm intestinal viromes
Source: ISME J. 2023 Apr 17;17(7):1004–14. doi: 10.1038/s41396-023-01408-x (PMC10284932; doi:10.1038/s41396-023-01408-x)
Supplement: Supplementary file 1 — Supporting texts 1-2, Supplementary figures 1-10 [file 41396_2023_1408_MOESM1_ESM.docx]

**Supporting Information**

**Benzene[a]pyrene stress impacts adaptive strategies and ecological functions of earthworm intestinal viromes**

Rong Xia^1,6^, Mingming Sun^1,6,*^, José Luis Balcázar^2,3^, Pingfeng Yu^4,*^ , Feng Hu^1^, Pedro J.J. Alvarez^5^

^1^ Soil Ecology Lab, Key Laboratory of Plant Immunity, Jiangsu Collaborative Innovation Center for Solid Organic Waste Resource Utilization & Jiangsu Key Laboratory for Solid Organic Waste Utilization, Nanjing Agricultural University, Nanjing 210095, China

^2^ Catalan Institute for Water Research (ICRA), Girona 17003, Spain

^3^ University of Girona, Girona 17004, Spain

^4^ College of Resource and Environmental Sciences, Zhejiang University, Hangzhou 310085, China

^5^ Civil and Environmental Engineering Department, Rice University, Houston 77005, USA

^6^ These authors contributed equally

^*^ Corresponding Author:

Email: sunmingming@njau.edu.cn (Mingming Sun), yupf@zju.edu.cn (Pingfeng Yu)

This supporting information includes 2 methods, 10 tables, and 10 figures.

**Supplementary texts**

**Text S1. Microcosm preparation and exposure experiments**

The collected soil samples without BaP contamination were air-dried at room temperature and sifted through a 2 mm sieve. The diatomite added with BaP was thoroughly mixed with the soil to yield a final concentration of 0, 0.1, 2.0, 20, and 200 mg of BaP kg^-1^ dry soil. Then all soils were equilibrated for seven days with water content of 15-20% [1].

The mature *Metaphire guillelmi* worms were acclimated for two weeks in the untreated soil under natural photoperiod and at 20 °C prior to the trials. After acclimatization, healthy adult earthworms with clear clitellum and weight of 5-6 g were chosen for the test [2]. After washing with sterile water, every 10 earthworms were transferred to the container containing 500 g soil [3]. The experiment consisted of a control and 4 treatments with 5 replicates of each treatment, giving a total of 25 containers. To ventilate and prevent the worms from escaping, the containers were covered with punctured lids. The culture containers were incubated at 20 °C in darkness for 28 days. The exposure time is determined according to The United States Environmental Protection Agency (USEPA) and the Organization for Economic Cooperation and Development (OECD) Protocol [4]. During the incubation period, dead earthworms were removed promptly, and water content was maintained at 15-20% [5].

**Text S2. The measurement of reactive oxygen species (****ROS) content and the activity of antioxidant enzymes**

The reactive oxygen species (ROS) content and the activity of antioxidant enzymes in earthworms were measured to assess the response of earthworms to different levels of BaP [6]. Earthworm intestinal tissue was powdered in a pre-cooled mortar and pestle, followed by adding potassium phosphate buffer (PBS, 0.05 M, pH 7.8) at the ratio of 1:10 (*w/v*) for homogenization in the ice bath. Homogenates were centrifuged at 8,000 g at 4 °C for 10 min to obtain supernatant for measurements [7]. Following the manufacturer’s instructions, the levels of ROS and three representative antioxidant enzymes, including superoxide dismutase (SOD), catalase (CAT), and peroxidase (POD), were detected by ROS-1-Y, SOD-1-W, CAT-1-W, and POD-1-Y kit (Suzhou Comin Biotechnology Co., Ltd., Suzhou, China), respectively [8]. ROS content and antioxidant enzyme activities were calculated based on the respective optical densities obtained by the microplate reader (PE Ensight). Specifically, ROS contents were expressed as the change in fluorescence units per second per g of tissue. SOD activity was expressed as U g^-1^ tissue, where U is the quantity of the enzyme that inhibited the xanthine oxidase coupling reaction by half. CAT activity was expressed as the enzyme activity catalyzing the degradation of 1 μmol H_2_O_2_ per g of tissue per minute. POD activity was expressed as U g^-1^ tissue, where U is a 0.01 change in absorbance per g of tissue per mL reaction mixture.

**Supplementary Tables**

**Table S1.** Characterization of lethality and bioconcentration of BaP in the culture soil and earthworm gut.

**Table S2.** Species alpha diversity of earthworm intestinal bacteria.

**Table S3.** Genes associated with BaP degradation and their relative abundance in the transcriptome.

**Table S4.** The score of a viral contig identified by VirSorter2 and DeepVirFinder.

**Table S5.** Species alpha diversity of earthworm intestinal phages.

**Table S6.** Phage-encoded AMGs identified by VIBRANT and DRAMV, and their functions annotation.

**Table S7.** The results of protein structure prediction by Phyre2.

**Table S8.** The name and corresponding symbol of the gene with the largest fold change between different samples and control.

**Table S9.** Phage - host predictions by CRISPR-match, tRNA match, and genome homology match.

**Table S10.** Lysogenic marker proteins (i.e., transposase, integrase, excisionase, resolvase, and recombinase) downloaded from Pfam.

**Supplementary Figures**


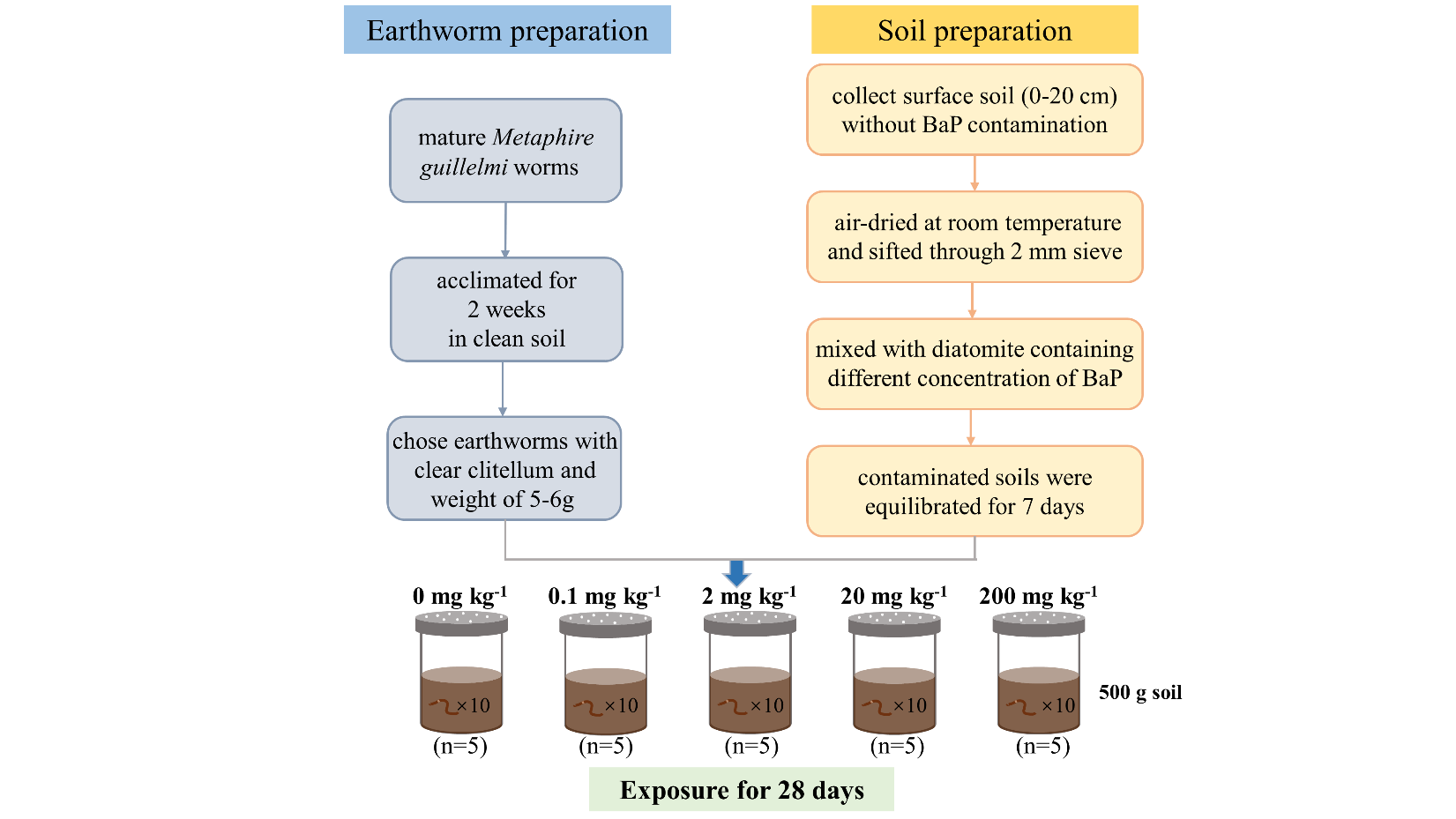


**Figure S1.** Concept diagram used to describe the experimental process of earthworm culture.


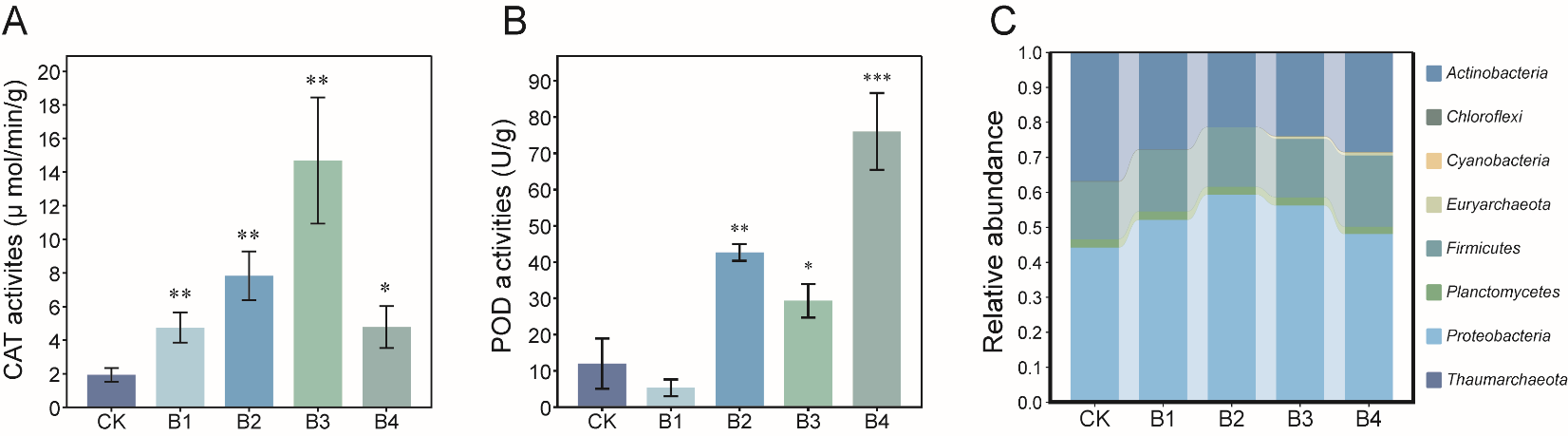


**Figure S2.** The activity of CAT (**A**) and POD (**B**) enzymes in earthworm gut after 28 days of exposure to BaP. (**C**) Relative abundance of earthworm intestinal bacteria at the phylum level.


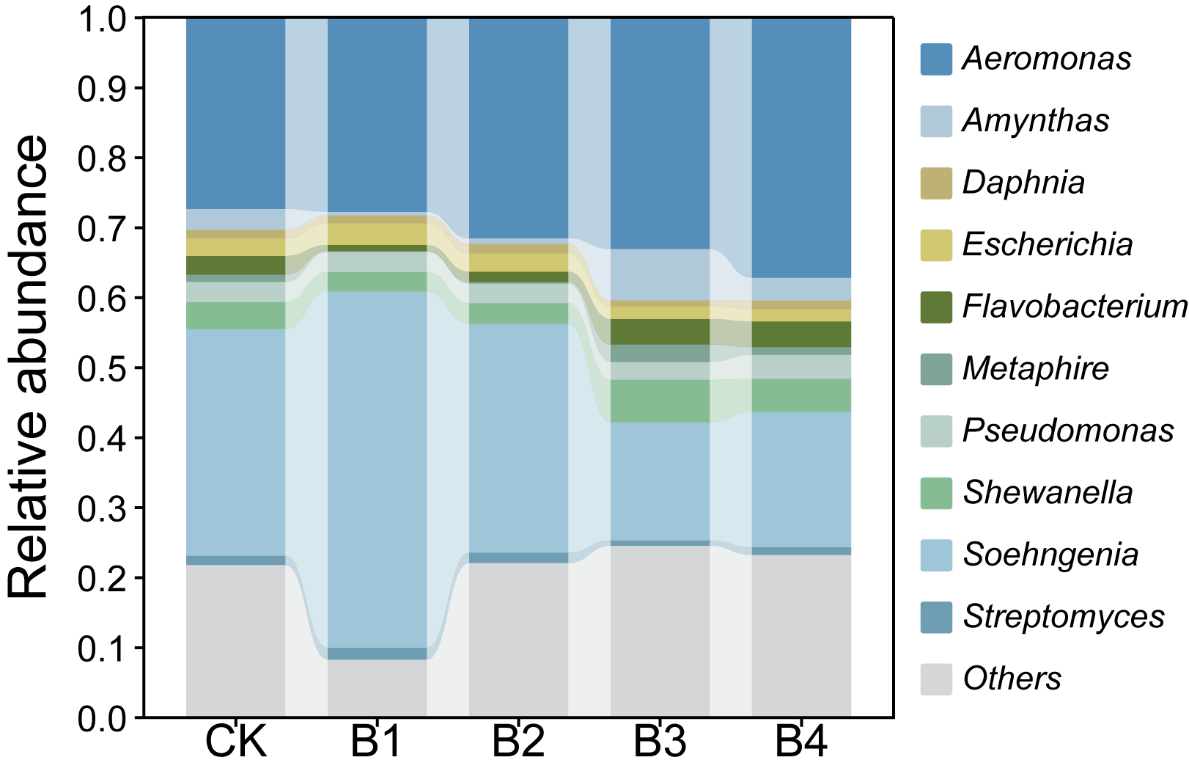


**Figure S3.** The functionally active microbial profile at genus level in earthworm intestines*.*


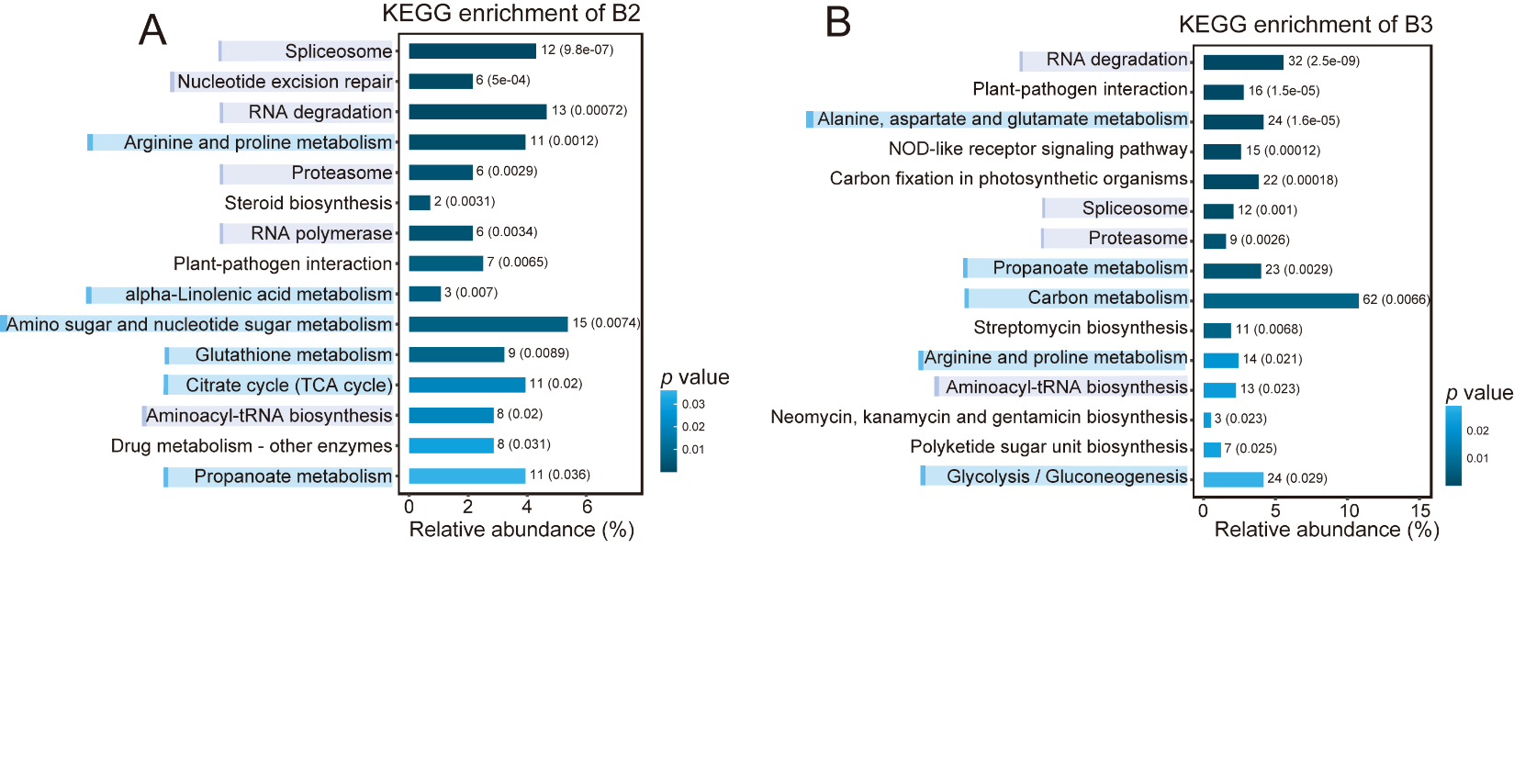


**Figure S4.** The histogram shows the top 15 enriched KEGG orthology (KO) pathways of upregulated genes under 2.0 (**A**) and 200 (**B**) mg kg^-1^ BaP exposure. The number of enriched upregulated genes in each pathway is labeled in front of the histogram, and the bars are ranked by *p* values.

**
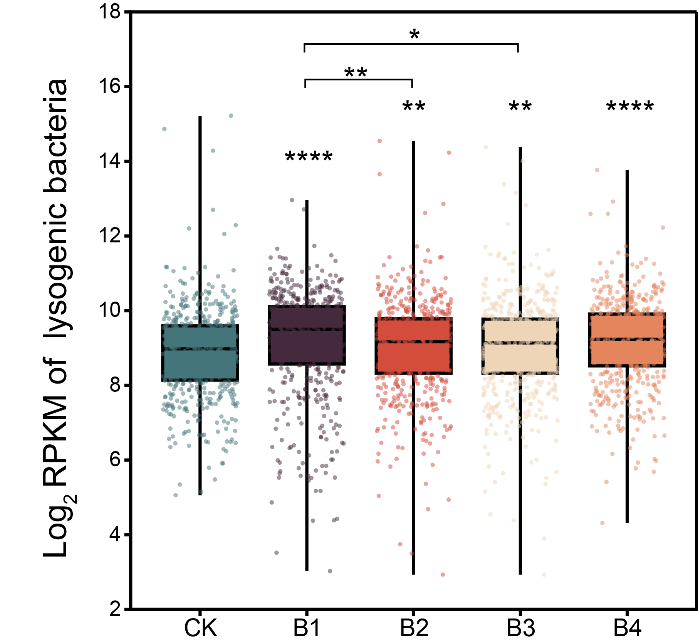
**

**Figure S5.** The abundance of lysogenic bacteria represented as Log10 RPKM.


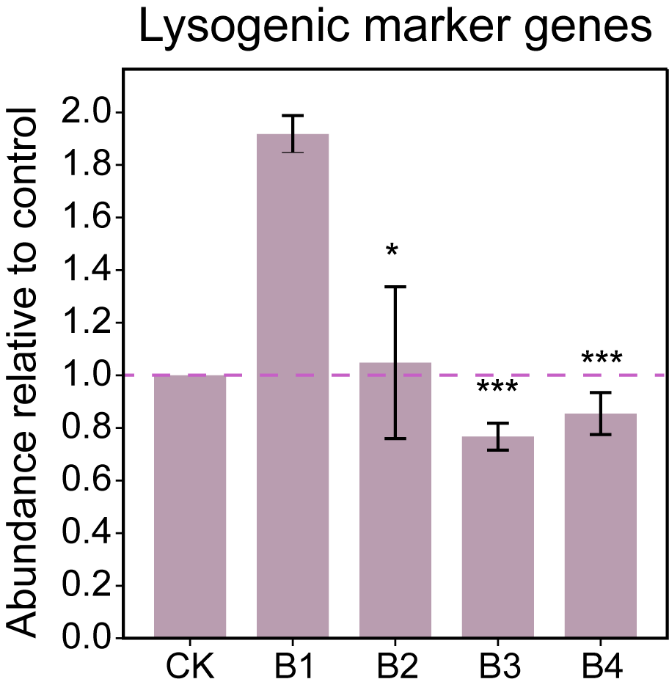
**Figure S6.** The regulation of lysogenic marker genes in metatranscriptomes relative to the control group.


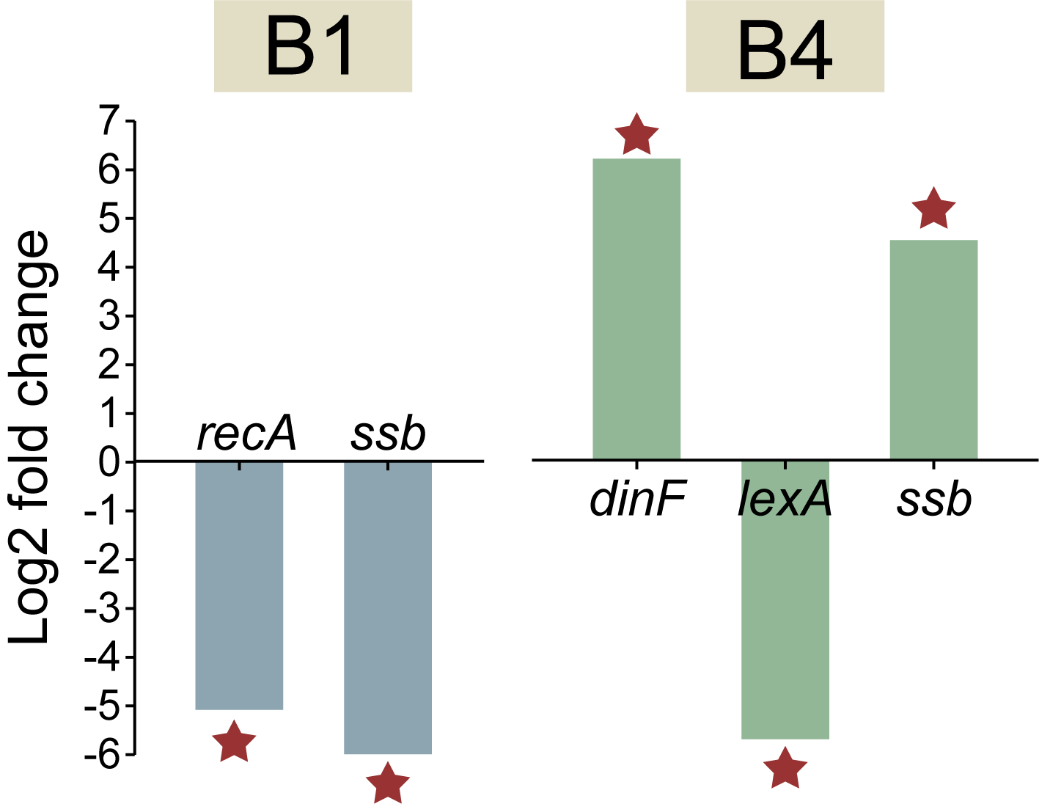


**Figure S7.** The log2 fold change of SOS response genes and repressor proteins’ abundance compared with CK. The red star indicates that the difference is significant.


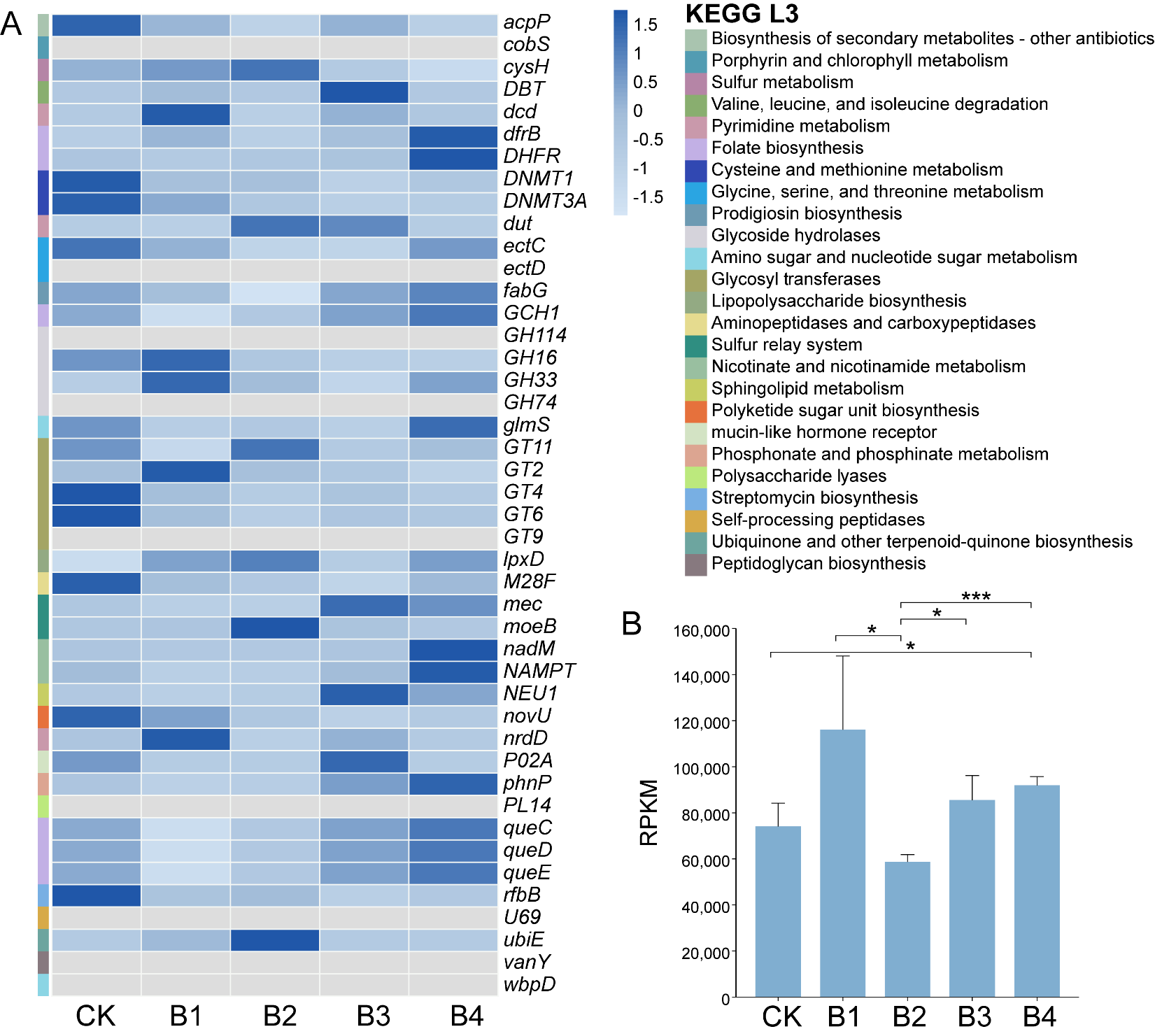


**Figure S8. (A)** The relative abundance of phage-carried AMGs in the metatranscriptome. The classification is according to KEGG L3 pathways. The abundance of zero is shown in gray. **(B)** The sum expression abundance (RPKM value) of phage-carried AMG in the metatranscriptome. * (*p* < 0.05) and ** (*p* < 0.01)


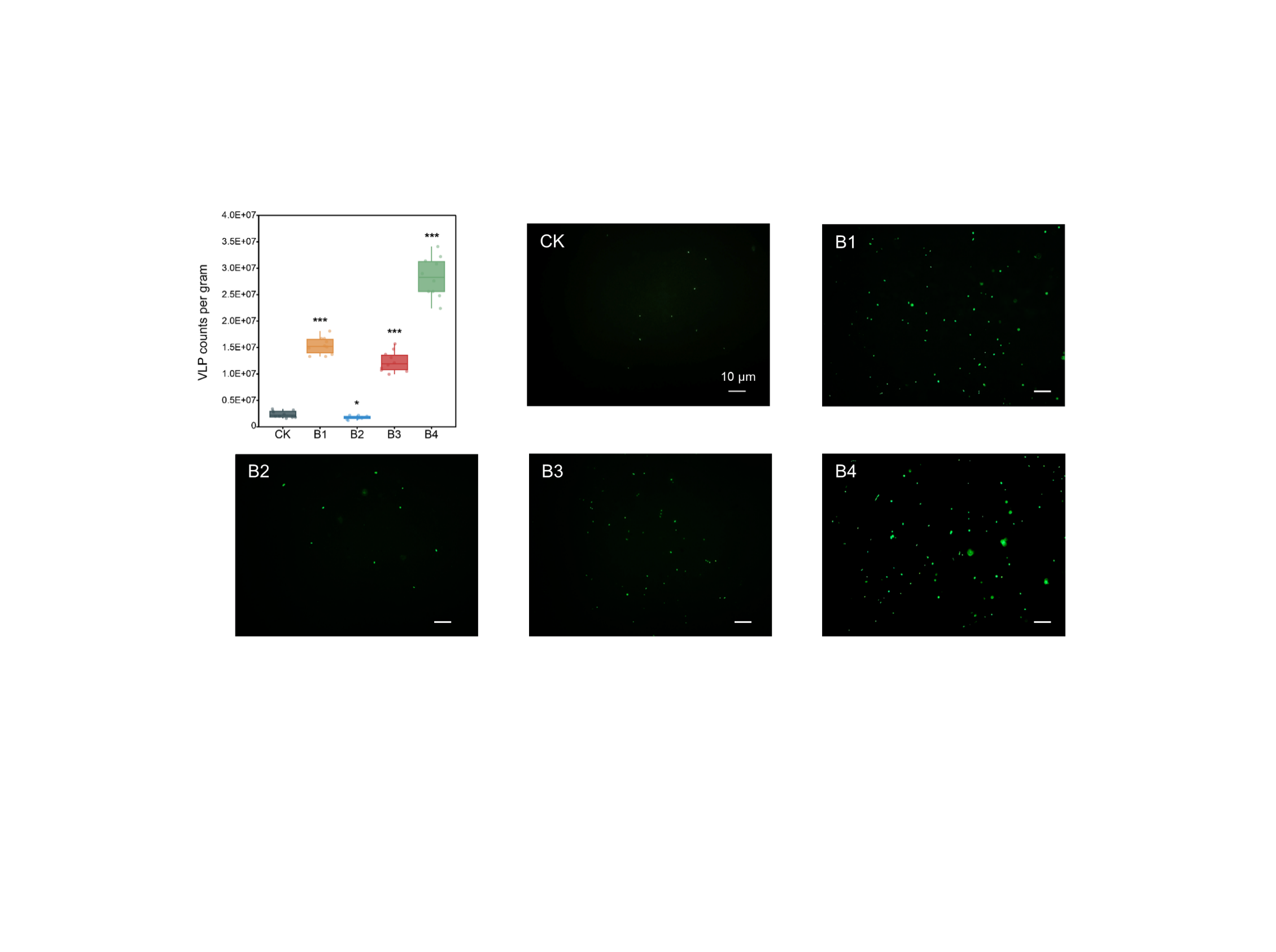


**Figure S9.** Count of VLP particles in the earthworm gut and the typical image phage fluorescence staining, in which phages are the most numerous tiny dots, while bacteria are larger with definite shapes and edges.


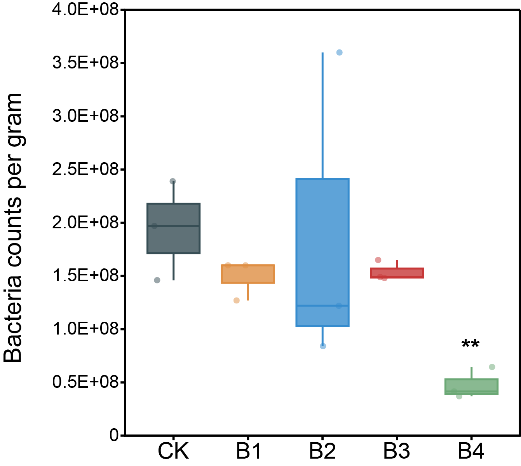


**Figure S10.** Count of bacteria in earthworm intestines by fluorescence quantitative PCR.

**References**

1. Wang X, Xia R, Sun M, Hu F. Metagenomic sequencing reveals detoxifying and tolerant functional genes in predominant bacteria assist Metaphire guillelmi adapt to soil vanadium exposure. J Hazard Mater. 2021;415:125666.

2. Zhu G, Du R, Du D, Qian J, Ye M. Keystone taxa shared between earthworm gut and soil indigenous microbial communities collaboratively resist chlordane stress. Environ Pollut. 2021;283:117095.

3. Zhang L, He N, Chang D, Liu X, Zhang X, Xu Y, et al. Does ecotype matter? The influence of ecophysiology on benzo[a]pyrene and cadmium accumulation and distribution in earthworms. Soil Biol Biochem. 2018;121:24-34.

4. Wang HT, Zhu D, Li G, Zheng F, Ding J, O'Connor PJ, et al. Effects of Arsenic on Gut Microbiota and Its Biotransformation Genes in Earthworm Metaphire sieboldi. Environ Sci Technol. 2019;53:3841-3849.

5. Yang Y, Liu P, Li M. Tri-n-butyl phosphate induced earthworm intestinal damage by influencing nutrient absorption and energy homeostasis of intestinal epithelial cells. J Hazard Mater. 2020;398:122850.

6. Wang H, Zhang X, Wang L, Zhu B, Guo W, Liu W, et al. Biochemical responses and DNA damage induced by herbicide QYR301 in earthworm (Eisenia fetida). Chemosphere. 2020;244:125512.

7. Qiao Z, Zhang F, Yao X, Yu H, Sun S, Li X, et al. Growth, DNA damage and biochemical toxicity of cyantraniliprole in earthworms (Eisenia fetida). Chemosphere. 2019;236:124328.

8. Chao H, Sun M, Ye M, Zheng X, Hu F. World within world: Intestinal bacteria combining physiological parameters to investigate the response of Metaphire guillelmi to tetracycline stress. Environ Pollut. 2020;261:114174.
